# Regulatory mechanisms of CH_4_ : air volume ratios on metabolic flux partitioning in methane-oxidizing bacteria and their impact on single cell protein biosynthetic efficiency

**DOI:** 10.3389/fmicb.2026.1646291

**Published:** 2026-01-22

**Authors:** Jianxiong Zhang, Jiao He, Jiaying Xin, Tianyu Cui, Chungu Xia

**Affiliations:** 1 Key Laboratory of Food Science and Engineering, Harbin University of Commerce, Harbin, China; 2 State Key Laboratory of Low Carbon Catalysis and Carbon Dioxide Utilization, Lanzhou Institute of Chemical Physics, Chinese Academy of Sciences, Lanzhou, China

**Keywords:** methane-oxidizing bacteria, single-cell protein, methane monooxygenases, gas-phase modulation, CH4, air, nitrogen fixation

## Abstract

**Introduction:**

C1 gas bioconversion for single-cell protein (SCP) production offers dual environmental benefits by mitigating greenhouse gases and generating protein resources. This study systematically determined optimal methane-to-air ratios (CH_4_:air, v/v) for enhancing methane-oxidizing bacteria (MOB) growth and SCP yield under three distinct nitrogen assimilation modes: nitrate-driven pMMO expression, ammonium-driven sMMO expression, and nitrogen-fixing sMMO expression.

**Methods:**

Experiments were conducted under three nitrogen assimilation regimes: nitrate-fed pMMO expression, ammonium-fed sMMO expression, and nitrogen-fixing sMMO expression systems. By adjusting the volumetric ratio of methane to air, the effects on bacterial growth, biomass accumulation, specific growth rate, and key enzymatic activities were evaluated. Measured parameters included OD_600_, cell dry weight, specific growth rate (μ_max_), and nitrogenase activity in the nitrogen-fixing system. Data from repeated measurements were subjected to statistical analysis to clarify the regulatory role of gas ratios on metabolic pathways.

**Results:**

In the nitrate-fed pMMO expression system, a CH_4_:air ratio of 1:3 yielded optimal growth, with an OD_600_ of 1.11, cell dry weight of 0.44 ± 0.023 g/L, and μ_max_ of 0.022 h^−1^. Similarly, the ammonium-fed sMMO expression system achieved best performance at the same ratio (OD_600_ 1.19, biomass 0.56 ± 0.014 g/L, μ_max_ 0.025 h^−1^). In contrast, the nitrogen-fixing sMMO expression system performed better at a lower oxygen ratio (CH_4_:air = 1:2), reaching an OD_600_ of 0.62, biomass of 0.28 ±0.008 g/L, nitrogenase activity of 1.09 nmol/(min mg protein), and μ_max_ of 0.016 h^−1^).

**Discussion:**

The results reveal oxygen's critical dual role: higher O_2_ levels enhance methane oxidation by activating the copper-dependent catalytic site of pMMO but simultaneously and irreversibly damage the oxygen-sensitive nitrogenase ssential for N_2_ fixation, suppressing its activity. Conversely, lower O_2_ protects nitrogenase but limits pMMO efficiency. This creates a fundamental metabolic trade-off where the optimal CH_4_/O_2_ ratio balances these opposing effects, strategically partitioning cellular energy either toward efficient methane assimilation (favored by higher O_2_) or toward the ATP-intensive process of nitrogen fixation (requiring lower O_2_). These identified gas-ratio thresholds provide actionable parameters for designing scaled SCP bioproduction systems, enabling effective coupling of industrial methane mitigation with sustainable protein synthesis through gas-phase engineering.

## 1 Introduction

Methane, the second most significant greenhouse gas after carbon dioxide, contributes to 46%−58% of anthropogenic emissions in the global atmospheric methane budget (Weindl et al., 2020). Among these sources, the oil and gas industry stand out as a major emitter, with methane releases substantially exacerbating the greenhouse effect (Saunois et al., 2020). Concurrently, global population growth and dietary shifts are driving surging demand for high-quality protein, while traditional aquaculture faces constraints due to limited fishmeal resources and environmental challenges in soybean meal production (Matassa et al., 2015). This necessitates the development of novel sustainable protein production methods (Dong et al., 2017; Teixeira et al., 2018). C1 gas bioconversion technology for single-cell protein (SCP) production presents a dual environmental benefit by simultaneously enabling greenhouse gas mitigation and protein resource generation.

Methane-oxidizing bacteria (MOB), as obligate methylotrophs, utilize methane/methanol as their sole carbon source through chemoheterotrophic metabolism (Lim et al., 2021; Shindell et al., 2012; Strong et al., 2015). Their core enzymatic machinery includes two structurally distinct methane monooxygenases (MMOs): the membrane-bound particulate MMO (pMMO) and the soluble MMO (sMMO), both regulated by Cu^2+^ ion gradients (Ward et al., 2018; Ross et al., 2019; Wang et al., 2020). Notably, MOB exhibit dual nitrogen assimilation capabilities: under standard cultivation, they preferentially utilize inorganic nitrogen sources (e.g., ammonium or nitrate), while under nitrogen-limiting conditions, certain strains activate nitrogenase systems to convert atmospheric N_2_ (78.1% abundance) into organic nitrogen (Auman et al., 2001). This metabolic flexibility not only reduces production costs but also aligns with the principles of sustainable biomanufacturing.

The gas-phase concentration gradients of key metabolic substrates (methane and oxygen) exert significant regulatory effects on MOB metabolic networks (Karthikeyan et al., 2015): (1) methane oxidation, as an obligate aerobic process, exhibits metabolic flux positively correlated with dissolved oxygen concentration (Hudspeth et al., 2024); (2) biological nitrogen fixation is oxygen-sensitive and requires microaerobic conditions (Boujenna and del Moral, 2021); (3) in nitrogen-rich media, methane oxidation dominates carbon metabolic flux, whereas under nitrogen starvation, metabolic flow shifts toward nitrogen fixation. This gas-dependent metabolic switching mechanism provides a theoretical foundation for optimizing SCP yield through gas ratio modulation in cultivation systems. Analytical data confirm that SCP not only matches conventional protein sources in nutritional composition (crude protein content: 60%−80%) but also enables valorization of methane emissions from oil and gas operations, offering combined carbon mitigation and resource recovery benefits (Nisbet et al., 2019; Owsianiak et al., 2022).

Building upon prior findings (highest methane utilization and SCP yields under nitrate nitrogen-pMMO expression, ammonium nitrogen-sMMO expression, and N_2_-sMMO expression), this study focused on elucidating the impact of CH_4_/air ratios on MOB growth kinetics and SCP biosynthesis efficiency. Implement gas-phase regulation (CH_4_/O_2_ volume ratios) is the primary control lever to optimize metabolic partitioning in methane assimilation processes. Couple nitrogen source selection with gas composition to achieve maximum biomass yield. These principles enable the development of gas-regulated SCP biorefineries that simultaneously mitigate industrial methane emissions and produce sustainable protein through carbon-nitrogen cycle integration.

## 2 Materials and methods

### 2.1 Medium preparation

Nitrate-Medium (NMS): the NMS medium consists of Solution A and Solution B: Solution A (per 1 L): 5.24 g K_2_HPO_4_·3H_2_O, 2.62 g KH_2_PO_4_, 0.04 g FeSO_4_·7H_2_O, 3.0 g MgSO_4_·7H_2_O, 3.0 g NaCl, 10.0 g KNO_3_ (nitrogen source), 0.0167 g FeCl_3_·6H_2_O, 0.151 g CaCl_2_. Solution B (per 1 L): 0.3 g MnSO_4_·H_2_O, 0.24 g NaMoO_4_·H_2_O, 0.34 g ZnSO_4_·7H_2_O. Mix 10 ml of Solution A and 0.1 ml of Solution B, then adjust to 100 ml with deionized water (Park et al., 2024).

Ammonium-Medium (AMS): the AMS medium similarly comprises Solution A and Solution B: Solution A (per 1 L): 5.24 g K_2_HPO_4_·3H_2_O, 2.62 g KH_2_PO_4_, 0.04 g FeSO_4_·7H_2_O, 3.0 g MgSO_4_·7H_2_O, 3.0 g NaCl, 5 g NH_4_Cl (nitrogen source), 0.0167 g FeCl_3_·6H_2_O, 0.151 g CaCl_2_. Solution B: Identical to NMS. Mix 10 ml of Solution A and 0.1 ml of Solution B, then adjust to 100 ml with deionized water.

Nitrogen-Free Medium (NFMS): the NFMS medium also consists of Solution A and Solution B. Solution A (per 1 L): 5.24 g K_2_HPO_4_·3H_2_O, 2.62 g KH_2_PO_4_, 0.04 g FeSO_4_·7H_2_O, 3.0 g MgSO_4_·7H_2_O, 3.0 g NaCl, 0.0167 g FeCl_3_·6H_2_O, 0.151 g CaCl_2_. Solution B: identical to NMS and AMS. Mix 10 ml of Solution A and 0.1 ml of Solution B, then adjust to 100 ml with deionized water.

Copper Supplementation for MMO Regulation (El Ghazouani et al., 2012). For pMMO expression: add 0.1 ml of filter-sterilized CuSO_4_ stock solution to achieve a final Cu^2+^ concentration of ~5 μmol/L. For sMMO expression: omit CuSO_4_ supplementation to maintain a Cu^2+^-free condition (0 μmol/L).

### 2.2 Cultivation of MOB

The *Methylosinus trichosporum* OB3b was used in the experiment, from Professor Xing Xinhui's research group at Tsinghua University, following the aforementioned protocols, 100 ml of NMS, AMS, and NFMS media were prepared in 250 ml culture flasks. The media were sterilized at 121 °C for 20 min using an autoclave and allowed to cool to room temperature before inoculation.

MOB seed culture was aseptically inoculated at 10% (v/v) of the medium volume, and the target OD_600_ for harvesting MOB seed culture is 0.8 ± 0.06. Subsequently, the headspace of each flask was purged and replaced with one of the following gas mixtures (balanced with air): CH_4_ : Air = 1:0 (100% methane); CH_4_ : Air = 1:1 (50% methane); CH_4_ : Air = 1:2 (33% methane); CH_4_ : Air = 1:3 (25% methane); CH_4_ : Air = 2:1 (67% methane); CH_4_ : Air = 3:1 (75% methane). Following inoculation and gas exchange, the flasks were incubated in a rotary shaker maintained at 30 °C and 180 rpm.

### 2.3 Analysis method

Methane consumption was directly determined and calculated using headspace gas chromatography under the following analytical conditions: a GC7900 gas chromatograph equipped with a combined column system (OV-101 capillary column and TDX-01 molecular sieve column) and a thermal conductivity detector was employed for effective separation of methane, carbon dioxide, oxygen, and nitrogen. The operating parameters included a column temperature of 50 °C, an injection port temperature of 100 °C, a detector temperature of 120 °C, hydrogen as carrier gas, and a operating current of 120 mA. Manual injections were performed using a 1 ml gas-tight syringe to sample the headspace of culture bottles. Standard curven was obtained by external standard calibration using a series of pure methane standards (0.1–1.0 ml). Cultivation followed a 24-h ventilation protocol over a 7-day period. Daily methane consumption was determined by measuring headspace methane via GC immediately after ventilation (pre-Tn) and 24 h later before the next ventilation (post-Tn). The daily consumption was calculated as the difference between the pre- and post-Tn methane volumes derived from the standard curve, and the total consumption was obtained as the cumulative sum over the 7-day cultivation period.

Every 24 h, 3 ml of bacterial culture was sampled, with distilled water serving as the blank control, with three sets of parallel repetitions for each condition and all data were analyzed using analysis of variance. The OD_600_ was measured using a UV-Vis spectrophotometer to assess microbial growth dynamics at different time points. By using the logistic function to fit the cell growth curve, the lag phase (λ) and maximum specific growth rate (μ_max_) of the bacterial cells under this condition can be obtained, and a dynamic mathematical model of the methane oxidizing bacterium *Methylosinus trichosporum* OB3b can be established. The formula equation is:


$$\begin{array}{l} \text{y}=\frac{A_{1}-A_{2}}{1+{\left(X/X_{0}\right)}^{P}}+A_{2} \end{array}$$ (1)

y represents OD_600_, A_1_ represents the initial concentration of the fermentation broth, A_2_ represents the maximum concentration of the fermentation broth, X represents the cell growth time, X_0_ represents the proportional constant of the maximum growth rate, and P represents the bacterial growth index.

After the completion of cell culture, collect 10 ml of bacterial solution and centrifuge at 4 °C and 8,000 r/min for 15 min to obtain bacterial cell precipitate. Rinse the cell precipitate twice with 0.05 mol/L phosphate buffer, and then add 2 ml of buffer to resuspend the precipitate. MMO activity was determined via the propylene epoxidation assay (Smith et al., 2011). Reaction setup: a 10 ml reaction vial was loaded with 2 ml of cell suspension and sealed. Using a disposable syringe, 4 ml of headspace air was removed and immediately replaced with 4 ml of premixed gas (propylene:air = 1:1 v/v). The vial was resealed and inverted for incubation in a rotary shaker (30 °C, 200 rpm) to facilitate epoxidation over 30 min. Reaction Termination: post-reaction, the vial was inverted and stored at 4 °C overnight to allow phase separation. The sample was then transferred to a 5 ml centrifuge tube and clarified by centrifugation (10,000 rpm, 15 min). The supernatant was collected for MMO activity analysis. Epoxide Quantification: propylene oxide (PO) concentration was measured via gas chromatography under the following conditions: column: BD-5HT capillary column (30 m × 0.25 mm × 0.25 μm). Temperatures: column: 170 °C (isothermal), Injector: 250 °C, flame ionization detector (FID) detector: 250 °C, Injection: manual, 0.5 μl splitless, retention time of PO: ~2.86 min. The calculation formula for MMO activity is as follows:


$$\begin{array}{l} {\text{MMO}}_{\text{active unite}}\;=\;\frac{{\text{C}}_{\text{epoxypropane }\times \text{ V}}}{\text{t }\times \;{\text{W}}_{\text{DCW}}} \end{array}$$ (2)

C_epoxypropane_: concentration calculated from the gas chromatographic peak area via the epoxypropane standard curve (nmol/ml); V: total volume of the reaction system (ml); t: reaction time (min); W_DCW_: dry cell weight (mg). Unit: nmol/(min·mg dcw), where “mg” refers to milligram of dry cell weight.

Nitrogenase Activity Assay via Acetylene Reduction Method (Montes-Luz et al., 2023). The nitrogenase activity was determined using the acetylene reduction assay (ARA). Briefly, 15 ml of bacterial culture was transferred into a 150 ml serum bottle, supplemented with 0.1% (v/v) methanol as an electron donor. The bottle was sealed with a butyl rubber stopper and aluminum crimp cap. For the reaction initiation: 10% of the headspace volume was removed using a disposable syringe. An equivalent volume of purified acetylene gas was immediately injected. The bottle was resealed to maintain gas-tight conditions. The reaction was carried out in a rotary shaker at 30 °C and 150 rpm for 60 min. Post-incubation, 1 μl of headspace gas was sampled using a gas-tight syringe and analyzed for ethylene production using a SCION456 gas chromatograph equipped with a flame ionization detector (FID). Chromatographic conditions were as follows: column: BD-5HT capillary column (30 m × 0.25 mm × 0.25 μm). Temperature program: column: 50 °C (isothermal), Injector: 200 °C, FID: 250 °C, Injection: manual split mode, 1 μl injection volume, Ethylene retention time: ~2.93 min. The calculation formula for Nitrogenase activity is as follows:


$$\begin{array}{l} {\text{Nitrogenase}}_{\text{active unite}}\;=\frac{{\text{C}}_{\text{ethylene }\times \text{ V}}}{{\text{t }\times \text{ W}}_{\text{DCW}}} \end{array}$$ (3)

C_ethylene_: concentration derived from the gas chromatographic peak area via the ethylene standard calibration curve (nmol/ml); V: headspace volume of the reaction vial (ml); t: reaction time (min); W_DCW_: dry cell weight (mg). Unit: nmol/(min·mg dcw), where “mg” refers to milligram of dry cell weight.

Determination of Cell Dry Weight (CDW) by Constant Weight Method. The cellular dry weight was measured using the constant weight method. Initially, cell pellets were collected via low-temperature centrifugation at 4 °C and 8,000 rpm for 15 min. The harvested biomass was subsequently washed twice with 0.05 M phosphate buffer (pH 7.0) to remove residual medium components. The processed cell pellets were then transferred to a constant-temperature drying oven and dehydrated at 70 °C until reaching constant weight. The dried samples were precisely weighed using an analytical balance with 0.1 mg resolution. For protein content analysis: (1) the lyophilized samples were reconstituted in 50 ml of distilled water. (2) Cell disruption was performed using an ultrasonic homogenizer under the following parameters: power output: 300 W, Pulsing cycle: 2 s ON, 4 s OFF, Total duration: 10 min. (3) The resulting cell lysate was collected for subsequent protein quantification. SCP Quantification via Bradford Assay: the SCP content was determined using the Coomassie Brilliant Blue G-250 dye-binding method (Yang et al., 2014). The assay principle relies on: formation of protein-dye complexes exhibiting maximum absorbance at 595 nm. Linear correlation between absorbance and protein concentration (0–1,000 μg/ml range). Colorimetric detection using a UV-Vis spectrophotometer. The SCP yield formula is as follows:


$$\begin{array}{l} Y_{SCP}=\frac{{△W}_{CDW}}{{△W}_{CH_{4}}} \end{array}$$ (4)

$$\begin{array}{l} {△W}_{CH_{4}}={\sum △}_{6}W_{CH_{4}} \end{array}$$ (5)

*Y*_*SCP*_ denotes the yield of SCP, Δ*W*_*CDW*_ represents the cell dry weight (g/L), and △*W*_*C*_*H*__4__ signifies the total amount of methane consumed during the cultivation process. ∑_△_6_*WCH*_4__represents the cumulative methane consumption across six ventilation intervals.

## 3 Results and discussion

### 3.1 Optimization of gas ratio of MOB during nitrate nitrogen-pMMO expression

**Figure 1:**
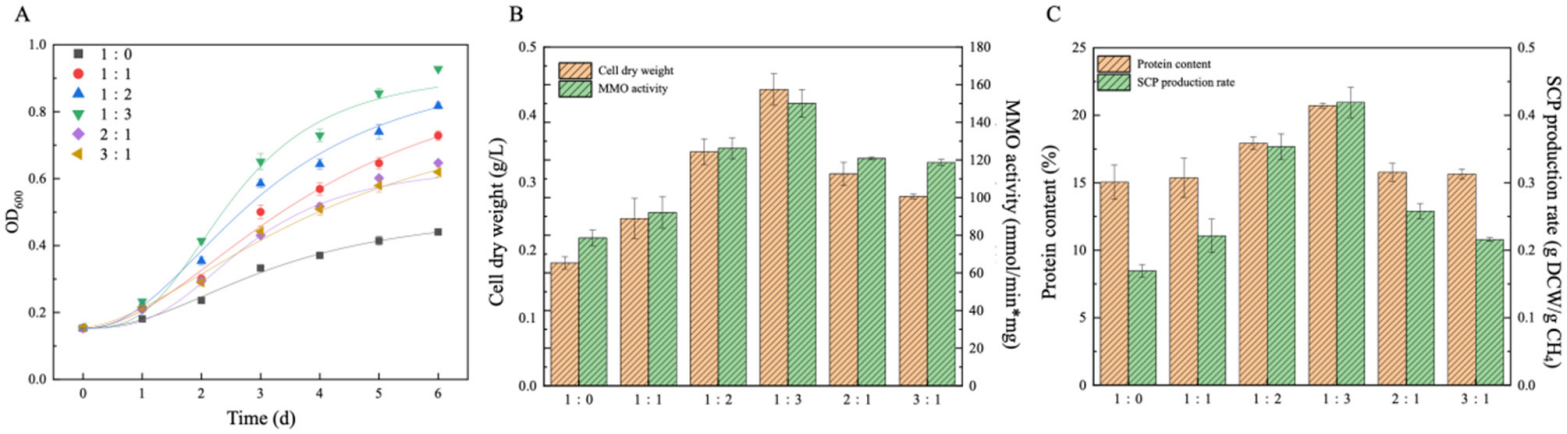
**(A)** Growth curve of MOB, **(B)** cell dry weight and MMO activity and **(C)** protein content and SCP production rate under different gas ratios during nitrate-nitrogen as nitrogen source and pMMO expression (the error bar represents the standard error).

Based on experimental data from Figure 1 and Table 1, the physiological metabolism of MOB was significantly modulated by gas-phase composition. Under CH_4_:air = 3:1 (v/v), the system exhibited suboptimal performance: maximum OD_600_ (0.63), cell dry weight (0.28 ± 0.004 g/L), and MMO-specific activity (118.72 nmol/(min·mg_dcw)) were all below optimal levels. This phenomenon arose from three metabolic constraints: (1) CO_2_ accumulation: from high methane metabolism induced proton motive force imbalance; (2) insufficient oxygen partial pressure (DO < 15% saturation) hindered copper cofactor binding in pMMO, reducing electron transport chain activity; (3) hypoxic microenvironments activated Fnr-regulated anaerobic metabolic compensation pathways, triggering energy diversion.

**Table 1:** Variation of maximum OD_600_, latency and maximum specific growth rate with different gas ratios (^*^p ≤ 0.05).

**Gas ratio**	**OD_600_**	**λ/h**	**μ_max_/h^−1^**
1:0	0.44	21.55 ± 0.31^*^	0.014
1:1	0.74	20.73 ± 0.33^*^	0.019
1:2	0.82	18.27 ± 0.24^*^	0.02
1:3	0.91	15.31 ± 0.3^*^	0.022
2:1	0.65	18.33 ± 0.36^*^	0.016
3:1	0.63	19.28 ± 0.37^*^	0.015

Comparative experiments revealed that in pure methane systems (CH_4_:air = 1:0), nitrate-dependent pMMO-expressing strains exhibited metabolic suppression (OD_600_ < 0.5). Incremental oxygen supplementation to CH_4_:air = 1:3 induced metabolic state transitions: (1) elevated DO enhanced cytochrome c oxidase-mediated respiratory phosphorylation efficiency; (2) Cu^2+^-assisted stabilization of pMMO tetrameric conformations; (3) biphasic gas-liquid mass transfer optimization yielded peak kinetic performance: maximum specific growth rate (μ_max_ = 0.022 h^−1^); CDW (0.44 ± 0.02 g/L) and soluble protein content (20.73 ± 0.18%) increased by 56.4% and 32.5%, respectively, compared to the 3:1 group.

Notably, the lag phase was reduced to 15.31 ± 0.3 h (representing a 26% reduction compared with the 3:1 group). Based on this observation, it is speculated that elevated oxygen levels promoted expression of the nitrogen assimilation gene cluster (nasAB-nrtP) through activation of the OmpR/PhoB two-component system, thereby facilitating rapid metabolic network reprogramming. These findings furnish key metabolic node parameters for implementing gas-phase regulation strategies in industrial-scale SCP production.

### 3.2 Optimization of gas ratio of MOB during ammonium nitrogen-sMMO expression

**Figure 2:**
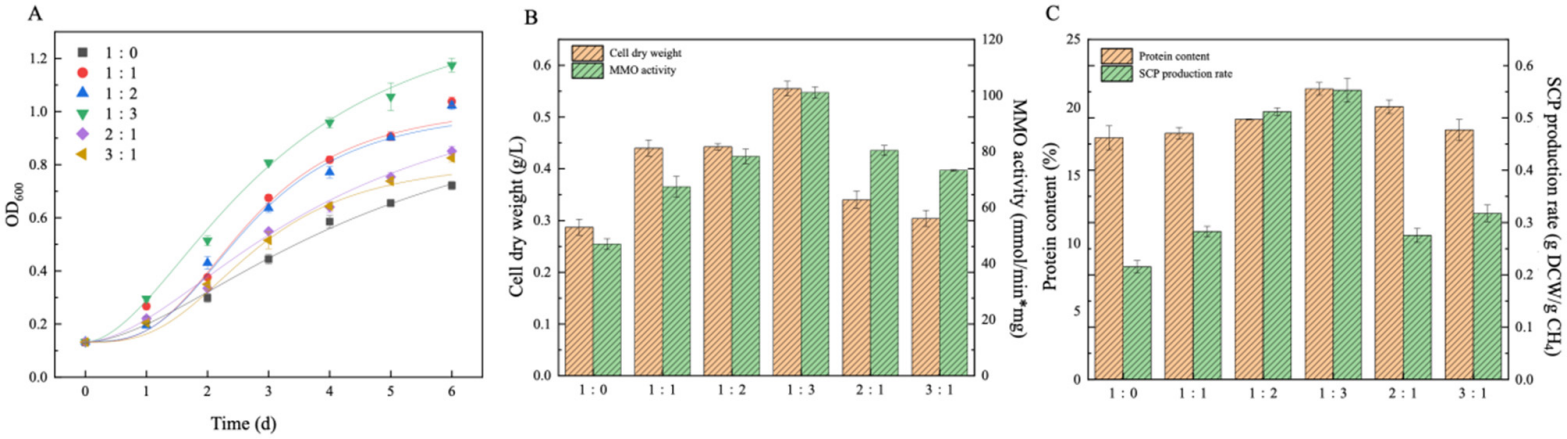
**(A)** Growth curve of MOB, **(B)** cell dry weight and MMO activity and **(C)** protein content and SCP production rate under different gas ratios during ammonium nitrogen as nitrogen source and sMMO expression (the error bar represents the standard error).

Comparative analysis of Figure 2 and Table 2 revealed that under strict anaerobic conditions (CH_4_:air = 1:0), the ammonium nitrogen-sMMO expressing MOB cultivation system exhibited significant metabolic inhibition. Specific manifestations included: biomass index OD_600_ reaching only 0.73, cell dry weight 0.29 ± 0.016 g/L, key enzymatic parameter MMO-specific activity declining to 46.99 nmol/(min·mg·dwc), and soluble protein content (17.78 ± 0.88%) being significantly lower than other treatment groups. This metabolic inhibition originated from impaired electron transport chain function in microaerophilic strains under hypoxic conditions, leading to reduced oxidative phosphorylation efficiency in sMMO-catalyzed methane hydroxylation and consequent impact on carbon assimilation flux. With gradual air supply introduction, elevated gas-phase oxygen partial pressure markedly activated the methane oxidation metabolic network. Under optimal CH_4_:air = 1:3 conditions, experimental groups demonstrated typical exponential growth phase characteristics: OD_600_ increased by 62.98% to 1.19, cell dry weight reached 0.56 ± 0.014 g/L, maximum specific growth rate (μ_max_) 0.025 h^−1^. Concurrently detected soluble protein content rose to 21.38 ± 0.46%, with lag phase shortened to 12.44 ± 0.31 h, indicating that oxygen's synergistic effects not only enhanced sMMO-mediated methane oxidation-assimilation flux but also optimized metabolic coupling efficiency between TCA cycle and amino acid synthesis pathways, increasing per-biomass SCP yield by 156%. This gas ratio-dependent metabolic regulation mechanism provides crucial theoretical foundation for industrial SCP production based on dynamic gas supply strategies.

**Table 2:** Variation of maximum OD_600_, latency and maximum specific growth rate with different gas ratios (^*^p ≤ 0.05).

**Gas ratio**	**OD_600_**	**λ/h**	**μ_max_/h^−1^**
1:0	0.73	18.31 ± 0.3^*^	0.018^*^
1:1	1.05	17.21 ± 0.31^*^	0.02^*^
1:2	1.03	15.02 ± 0.23^*^	0.023^*^
1:3	1.19	12.44 ± 0.31^*^	0.025^*^
2:1	0.86	15.08 ± 0.35^*^	0.018^*^
3:1	0.82	16.35 ± 0.35^*^	0.018^*^

### 3.3 Optimization of gas ratio of MOB during N_2_-sMMO expression

**Figure 3:**
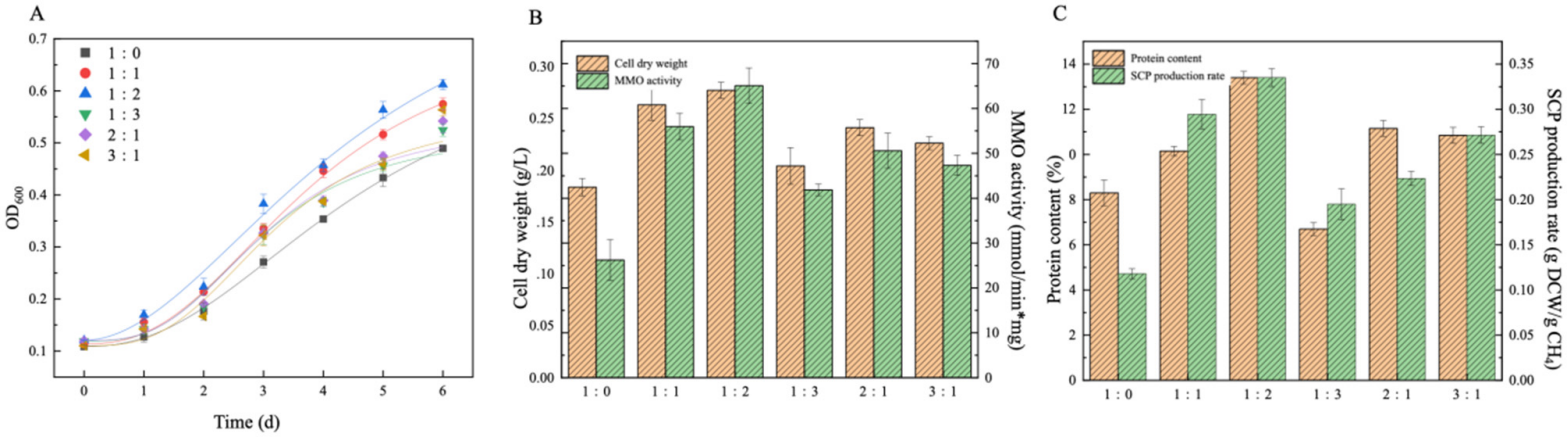
**(A)** Growth curve of MOB, **(B)** cell dry weight and MMO activity and **(C)** protein content and SCP production rate under different gas ratios during N_2_ fixation as nitrogen source and sMMO expression (the error bar represents the standard error).

Systematic analysis of Figure 3 and Table 3 reveals the underlying mechanisms of gas composition effects on methanotrophic bacterial metabolism regulation. In pure methane systems (CH_4_:Air=1:0), nitrogen-dependent sMMO-expressing strains exhibited the lowest specific growth rate (μ = 0.013 h^−1^) due to dual constraints: firstly, MMO enzymatic kinetics became DO-limited under strictly anaerobic conditions, significantly reducing methane oxidation metabolic flux; secondly, undetectable nitrogenase activity indicated nitrogen assimilation pathway blockage forming a growth-limiting bottleneck.

**Table 3:** Variation of maximum OD_600_, latency and maximum specific growth rate with different gas ratios (^*^p ≤ 0.05).

**Gas ratio**	**OD_600_**	**λ/h**	**μ_max_/h^−1^**	**Nitrogen fixation content (mmol/L)**
1:0	0.49	30.89 ± 0.33^*^	0.013	—
1:1	0.58	27.15 ± 0.22^*^	0.016	0.009
1:2	0.62	26.49 ± 0.3^*^	0.016	0.015
1:3	0.52	29.02 ± 0.29^*^	0.014	0.014
2:1	0.53	27.92 ± 0.37^*^	0.015	0.011
3:1	0.56	28.51 ± 0.3^*^	0.015	0.008

With air introduction (CH_4_:Air=1:3), while elevated dissolved oxygen enhanced methane oxidation rate, excessive oxygen partial pressure caused irreversible oxidative damage to nitrogenase complexes, reducing nitrogen fixation efficiency. This directly manifested in physiological parameters: OD_600_ = 0.52, cell dry weight 0.21 ± 0.018 g/L, and soluble protein content 6.7 ± 0.28%, significantly lower than other groups.

Optimal performance occurred at CH_4_:Air = 1:2, where precise DO control enabled metabolic flux redistribution: maintaining sMMO's optimal redox potential while creating microaerobic conditions to protect nitrogenase active sites. Experimental data showed: maximum specific growth rate reached 0.016 h^−1^, lag phase shortened to 26.49 ± 0.3 h, OD_600_ = 0.62 and cell dry weight 0.28 ± 0.008 g/L, and soluble protein content increased to 13.4 ± 0.28%. This discovery provides critical parameters for industrial SCP production optimization, suggesting dynamic gas regulation to balance MMO catalytic efficiency and nitrogenase stability can overcome substrate competition inhibition in conventional cultivation systems.

Although the closed batch culture system used in this study did not monitor gas consumption or supplement reaction gases in real time, the core objective of the experimental design is to establish a benchmark correlation between initial gas ratios and metabolic responses under different nitrogen source modes (such as growth kinetics parameters, enzyme activity thresholds, etc.). Although the gas phase ratio undergoes dynamic changes during the reaction process, all comparative experiments maintain consistent closed conditions, thus obtaining the optimal initial ratio threshold with clear comparative value.

## 4 Conclusion

This study demonstrated significantly superior growth rates, cell mass, and methane oxidation efficiency in MOB under nitrate (pMMO expression) or ammonium (sMMO expression) conditions with CH_4_:air (1:3), compared to a nitrogen-fixing (sMMO expression) system using N_2_. This difference arises because N_2_ fixation via the nitrogenase complex consumes substantial cellular energy (16 ATP per N_2_ molecule), diverting resources from carbon assimilation during methane oxidation. Furthermore, the elevated oxygen levels in the CH_4_:air (1:3) system created a metabolic paradox: while promoting pMMO activity via its copper domain, excessive oxygen irreversibly damaged the oxygen-sensitive nitrogenase, reducing fixation rates. An optimal dissolved oxygen threshold was therefore essential for simultaneously maximizing methane monooxygenase (MMO) activity and preserving nitrogenase function through microaerobic conditions. The results confirm that the CH_4_/O_2_ ratio acts as a dual regulator, providing substrates while modulating metabolism to collectively determine MOB's metabolic phenotype and single-cell protein production efficiency, offering a theoretical basis for high-density cultivation strategies using coupled nitrogen source and gas phase control.

## Data Availability

The raw data supporting the conclusions of this article will be made available by the authors, without undue reservation.
